# Obituary

**Published:** 1889-01

**Authors:** 


### Obituary.

JAMES N. MATTHEWS, editor, proprietor, and publisher of the Buffalo *Express*, after several weeks' illness, finally yielded to the "grim messenger" on Thursday, December 20, 1888, breathing out his life at his residence in this city, surrounded by his devoted family and friends. Mr. Matthews has been identified with the printing art in Buffalo since 1845. He has been largely instrumental in developing it from the simplicity of that period to the great enterprises that are now carried on here, and which have made Buffalo famous as a printing center. Few men were his equals in the knowledge of all that makes printing substantial and beautiful, and none his superiors. As an editor, he was better known to a later generation, but, as a printer, his name will be ever endeared to the followers of that art. The cover of this JOURNAL was a suggestion of his, and he informed the writer not long ago that he worked on its first numbers as a compositor, stating that it was with great difficulty that he mastered the "copy" of its former editor, the late Dr. Austin Flint. This is not the place for an extended notice of this remarkable man—one worthy of his character and deeds—but we cannot refrain from paying a slight tribute of respect to his memory—from placing one little flower on his passing bier The story of his life cannot be more beautifully told than in the words of the editor of the *Express* in closing the brief announcement of Mr. Matthews's death next morning. We reproduce the touching metaphor as a feeling outburst of lofty eloquence:

"Man of fire and dew, farewell! Your spirit was twenty, your years were sixty, but—alas!—your body was eighty. Farewell!"

